# Effects of a protein‐restricted diet on body weight and serum tyrosine concentrations in patients with alkaptonuria

**DOI:** 10.1002/jmd2.12255

**Published:** 2021-11-09

**Authors:** Birgitta Olsson, Lakshminarayan Ranganath, Jean‐Baptiste Arnoux, Richard Imrich, Anna Milan, Mattias Rudebeck

**Affiliations:** ^1^ Swedish Orphan Biovitrum AB (publ) Stockholm Sweden; ^2^ Institute of Ageing & Chronic Disease University of Liverpool Liverpool UK; ^3^ Departments of Clinical Biochemistry and Metabolic Medicine Liverpool University Hospitals NHS Foundation Trust (LUH) Liverpool UK; ^4^ Hôpital Necker‐Enfants Malades Paris Cedex 15 France; ^5^ Institute of Clinical and Translational Research, Biomedical Research Center, Slovak Academy of Sciences Bratislava Slovakia; ^6^ National Institute of Rheumatic Diseases Piešťany Slovakia

**Keywords:** Alkaptonuria, body weight, diet, keratopathy, protein, tyrosine

## Abstract

In an open‐label, controlled study of nitisinone in alkaptonuria (SONIA 2), patients were advised to lower dietary protein intake to reduce serum tyrosine (s‐Tyr) levels and the risk of keratopathy. A body weight increase was observed in the nitisinone‐treated patients but not in the control group. To investigate the effectiveness and consequence of protein restriction in patients with alkaptonuria, a post‐hoc analysis of SONIA 2 was performed. One hundred and thirty‐eight patients were randomised (nitisinone: n = 69, controls: n = 69). Comparison of baseline and Month 12 data on 24‐h urinary excretion of HGA (u‐HGA_24_) and urea (u‐urea_24_, used as an approximate protein intake measure), tyrosine and body weight were performed using paired *t* tests. Comparisons of data between groups were made using 2‐sample *t* tests. We found that u‐urea_24_ decreased more in nitisinone‐treated than controls. The study centre with lowest average s‐Tyr and u‐urea_24_ (nitisinone arm) at Month 12 also had lowest keratopathy incidence (3.1%), while the centre with highest values showed the highest (14.6%). S‐Tyr was generally high in those with keratopathy, but those without keratopathy had similar elevated values. A similar pattern across centres was seen for body weight changes, with a statistically significant weight increase in nitisinone‐treated patients at centres with lower u‐urea_24_ values. Therefore, in nitisinone‐treated patients, protein restriction led to increased body weight but may also have lowered the risk of developing keratopathies. If introduced, a protein‐restricted diet should be supervised by a dietician and, when appropriate, include amino acid supplements deficient in tyrosine and phenylalanine, to avoid malnutrition and undesired weight increase.

**SYNOPSIS**

In patients with AKU treated with nitisinone, a protein‐restricted diet may reduce the risk of keratopathy, but without dietician supervision this could lead to an increase in body weight.

## 1 INTRODUCTION

Alkaptonuria (AKU, OMIM reference 203500) is a rare autosomal recessive disorder caused by a deficiency in homogentisate 1,2‐dioxygenase (HGD, EC 1.13.11.5), the third enzyme in the tyrosine catabolic pathway. HGD converts homogentisic acid (HGA, 2,5‐dihydroxyphenylacetic acid) to 4‐maleylacetoacetic acid. Inability to metabolise HGA leads to urinary excretion of at least 90% of the compound, or 3‐6 g per day, in patients with AKU.^1^, ^2^, ^3^, ^4^ Despite this pronounced renal elimination, some HGA is oxidised to a melanin‐like pigment, via benzoquinone acetic acid, which is deposited in connective tissue, especially in cartilage, a process called ochronosis.^5^ Ochronosis leads to early onset arthritis of the spine and synovial joints and other debilitating symptoms.^6^, ^7^

Nitisinone (NTBC or 2‐(2‐nitro‐4‐[trifluoromethyl]benzoyl)cyclohexane‐1,3‐dione), is a potent competitive inhibitor of the enzyme 4‐hydroxyphenyl‐pyruvate dioxygenase (HPPD, EC 1.13.11.27) which converts 4‐hydroxyphenylpyruvate to HGA. A 4‐year open‐label, randomised, controlled study in 138 patients with AKU (‘SONIA 2’) found nitisinone 10 mg once daily to decrease ochronosis and disease progression in these patients^8^ by decreasing HGA formation, as evidenced by decreased urinary excretion of HGA by on average 99.7%.

Because of its mode of action, treatment with nitisinone leads to increased circulating tyrosine. This is in turn known to cause tyrosine crystallisation in the cornea leading to dendritiform keratopathy with symptoms such as red eyes, tearing and eye pain.^9^ To reduce this risk of keratopathy, patients in SONIA 2 were advised to lower their daily dietary intake of protein, however without a supervised dietary intervention.

One observation in the study was that patients in the nitisinone group showed an average (SD) weight increase over the 4 years of 2.9 (4.8) kg while the change in the control group was only 0.2 (4.0) kg.^8^ The reasons for this weight gain in nitisinone‐treated patients were not explored, as it was beyond the scope of the initial analysis.

We present here additional data from the SONIA 2 study (NCT01916382), examining the effectiveness of the unsupervised low‐protein strategy in terms of preventing corneal keratopathy, the compliance with this protein diet, and the effects of the protein‐restricted diet in both nitisinone‐treated patients and untreated controls in terms of body weight and metabolic analyses.

## 2 MATERIALS AND METHODS

### 2.1 Patients

Eligible patients were at least 25 years old, had increased urinary excretion of HGA compared to normal subjects and at least some sign of disease progression, such as visible ochronosis, back or joint pain or other manifestations of AKU. Detailed inclusion and exclusion criteria have been presented earlier.^8^

Patients were treated at three centres in Europe. Centre 1 (Liverpool, UK) treated 41 patients from western Europe (excl. the UK), from Spain and Italy to Scandinavia. Centre 2 (Piešťany, Slovakia) treated 65 patients of whom the majority were from Slovakia with a few patients from other countries in eastern Europe, and approximately 20 patients from Jordan. Centre 3 (Paris, France) treated 32 patients mainly from France but with a few patients from Belgium.

### 2.2 Study design and treatments

SONIA 2 was an international, multicentre, open‐label, evaluator‐blinded, parallel‐group, randomised study in 138 patients with AKU, of whom 69 received 10 mg nitisinone once daily for up to 4 years and 69 patients served as an untreated control group. There were six visits to the study centres, including baseline and yearly visits up to Month 48, with an additional safety follow‐up at Month 3.

Studies in AKU cannot be blinded, as oxidised HGA turns the urine dark, and patients can easily see whether they receive nitisinone treatment or not. All patients were thus aware of their treatment arm assignment.

Nitisinone‐treated patients who developed keratopathies, caused by high serum tyrosine (s‐Tyr) levels, could continue in the study on a dose of 2 mg/day, after temporary withdrawal of nitisinone and full recovery of the event.

All patients were given dietary advice, aiming at reducing protein intake. In particular, they were instructed to eat less meat and fish. They were informed that the reason was to reduce s‐Tyr levels and the risk of negative effects on the cornea (keratitis, keratopathy) in the nitisinone‐treated patients. At Centres 1 and 2, the advice was given by the investigators. At Centre 3, a dietician met the patients and discussed the diet, which aimed at a daily protein intake of 0.8 g/kg/day (the written diet instruction is presented in the Supplement).

### 2.3 Measurements

Measurements of serum HGA (s‐HGA) and s‐Tyr were performed in pre‐breakfast samples (pre‐dose in treated patients) at each visit to the clinic. Measurements of 24‐h urinary excretion of HGA (u‐HGA_24_) and urea (u‐urea_24_, used here as a proxy measure of protein intake) were performed in urine collected during 24 h at the clinic visits.

The protocol did not stipulate when the serum samples were to be collected relative to the urine collection period. At Centre 1, the serum was sampled before starting urine collection and at Centre 3 after its completion. At Centre 2, serum was collected before start of urine collection at baseline but after urine collection at Month 12.

HGA and tyrosine in serum and urine were determined by two earlier presented liquid chromatography–mass spectrometry methods.^10^, ^11^ Urine urea was assayed photometrically using an automated assay (hydrolysis with urease and subsequent oxidation of NADH) on a Roche Cobas 701.

All patients, including controls, underwent slit‐lamp examinations at all visits to the clinics to check for possible development of keratopathies. Patients were further instructed to contact the investigator in case of any ocular symptoms. When this occurred, slit‐lamp examinations were performed also between scheduled visits.

### 2.4 Calculations and statistics

To investigate how the change in diet affected HGA, tyrosine, urea, and body weight, only baseline and Month 12 data (M12 dataset) were used. These provide the most reliable pharmacology data, given the long‐term design of our study with a greater number of withdrawn patients at later visits. Only patients with data at both baseline and Month 12 were therefore included in these calculations. One patient who used a nitisinone dose of 2 mg/day at Month 12 was also excluded from the dataset.

Comparisons of data at baseline and Month 12 were performed using paired *t* tests, and comparisons of data between groups of patients were made using 2‐sample *t* tests. Statistical significance was defined as a *p*‐value <0.05.

The comparison of tyrosine and urea in patients who developed keratopathies with those who did not, used all data collected during the study. The same applies to tyrosine and HGA data in patients who reduced the dose to 2 mg/day after developing keratopathies. However, these data are only presented with descriptive statistics.

## 3 RESULTS

### 3.1 Demographics

Of the 129 patients included in the M12 dataset, 66 were treated with nitisinone 10 mg/day and 63 were in the untreated control group. Demographic data are given in Table S1. Approximately 64% of the patients were overweight or obese, with relatively more obese, and fewer normal weight, patients in the nitisinone group compared to controls (Table S2).

### 3.2 Changes in protein intake and body weight post‐baseline

One year after the introduction of the dietary advice given at the baseline visit, there was a statistically significant decrease in u‐urea_24_ by an average (SD) of 31.3 (89.6) mmol, or 6.28 (28.63) % in the full M12 dataset. There was, however, notable differences between the three centres; Centre 1 showed no change in u‐urea_24_, while decreases from baseline of 8.69 (27.07) and 15.20 (27.34) % were seen at Centres 2 and 3, respectively, both statistically significant (Table S3).

When the data was divided by treatment group, there was no difference between the two groups at Centre 1, while at Centres 2 and 3 the decrease in u‐urea_24_ was almost twice as large in the nitisinone‐treated patients as in the untreated controls. However, this numerical difference between the two treatment groups was not statistically significant (Table 1).

**TABLE 1:** Mean (SD) changes in u‐urea_24_ from baseline to Month 12, by treatment and centre

Control					Nitisinone				
Centre (n)	Baseline (mmol)	Month 12 (mmol)	Change (mmol)	Change (%)^a^	Centre (n)	Baseline (mmol)	Month 12 (mmol)	Change (mmol)	Change (%)^a^
1 (16)	324.2 (103.4)	324.7 (84.8)	0.5 (90.2)	5.51 (30.11)	1 (18)	313.9 (93.5)	321.5 (69.3)	7.5 (77.5)	7.93 (28.64)
2 (32)	326.7 (92.7)	296.6 (100.1)	−30.15 (92.41)	−5.97 (29.79)	2 (30)	330.5 (97.1)	279.3 (75.5)	−51.3 (93.0)	−11.59 (24.00)
3 (14)	286.7 (76.7)	250.6 (97.7)	−36.1 (90.0)	−10.14 (29.99)	3 (16)	281.0 (74.8)	220.3 (72.4)	−64.7 (75.9)	−20.94 (24.92)
Total (62)	317.0 (92.4)	293.4 (97.9)	−23.6 (91.0)	−3.95 (30.00)	Total (64)	315.5 (91.9)	276.4 (81.0)	−37.1 (88.0)	−8.11 (27.34)

*Note*: *p*‐values, absolute change in control group: Overall: 0.045; Centre 1: 0.984; Centre 2: 0.075; Centre 3: 0.158. *p*‐values, absolute change in nitisinone group: Overall: 0.001; Centre 1: 0.684; Centre 2: 0.005; Centre 3: 0.006. *p*‐value for absolute change in controls vs absolute change in nitisinone group: Overall: 0.400.

^a^ Based on individual % changes.

A similar pattern is seen for the changes in body weight. There was no change from baseline to Month 12 at Centre 1, while there were statistically significant increases in weight at Centres 2 and 3. These latter two centres had the patients with the highest and lowest weights at baseline, respectively (Table S3). When the body‐weight data was stratified by treatment, the control group showed no change in weight, except for a significant decrease at Centre 1. For the nitisinone‐treated patients, a statistically significant weight increase was seen overall and at Centres 2 and 3, and with a trend towards an increase even at Centre 1 (Table 2).

**TABLE 2:** Mean (SD) changes in body weight from baseline to Month 12, by treatment and centre

Control					Nitisinone				
Centre (n)	Baseline (kg)	Month 12 (kg)	Change (kg)	Change (%)^a^	Centre (n)	Baseline (kg)	Month 12 (kg)	Change (kg)	Change (%)^a^
1 (16)	68.9 (11.1)	67.1 (10.1)	−1.8 (−2.4)	2.95 (3.97)	1 (18)	74.3 (13.8)	75.8 (14.1)	1.5 (3.6)	2.06 (5.08)
2 (32)	80.7 (15.9)	81.5 (15.2)	0.8 (1.2)	4.39 (4.65)	2 (32)	78.4 (13.9)	81.1 (14.2)	2.7 (2.7)	3.53 (3.35)
3 (15)	66.6 (13.8)	66.8 (14.7)	0.2 (0.1)	2.43 (3.51)	3 (16)	69.4 (16.6)	72.8 (18.4)	3.3 (4.9)	4.76 (6.15)
Total (63)	74.3 (15.6)	74.3 (15.6)	0.0 (3.8)	0.04 (4.43)	Total (66)	75.5 (15.1)	78.0 (15.5)	2.50 (3.42)	3.45 (4.31)

*Note*: *p*‐values, change in control group: Overall: 0.973; Centre 1: 0.027; Centre 2: 0.322; Centre 3: 0.754. *p*‐values, change in nitisinone group: Overall: <0.001; Centre 1: 0.097; Centre 2: <0.001; Centre 3: 0.017. *p*‐value for absolute change in controls vs absolute change in nitisinone group: 0.0002.

^a^ Based on individual % changes.

A plot of individual changes in body weight vs changes in u‐urea_24_ for the full M12 dataset shows no correlation between these two variables, nor for changes in s‐Tyr vs u‐urea_24_. On the other hand, good correlations were seen for u‐HGA_24_ vs u‐urea_24_ as well as for s‐HGA vs u‐urea_24_ (Figure S1).

The effect of diet on HGA and tyrosine can only be studied in untreated patients, since nitisinone has a very pronounced effect on these through its inhibition of HPPD. In untreated patients, u‐HGA_24_ decreased statistically significantly by an average of 6729 μmol (8.02%) from baseline to Month 12 (Table S4). There were no statistically significant effects on s‐HGA and s‐Tyr. Numerically, there was a slight decrease (3.29%) in s‐Tyr, but a 9.30% increase in s‐HGA (Tables S5 and S6).

### 3.3 Tyrosine and risk of developing keratopathy

Nine patients, all in the nitisinone group, spontaneously reported signs of keratopathy (dry eyes, eye pain, blurred vision, tearing). Of these, eight cases were subsequently confirmed by a slit‐lamp examination, and one more case was found by examination at a pre‐planned visit. One of the spontaneously reported suspected keratopathies could not be confirmed as the patient was unable to travel to the study centre. The first symptoms in any patient appeared after less than 3 months (83 days) of nitisinone treatment and the last one after about 2.9 years. After that, no additional patients reported any signs of keratopathy (Table S7).

No clear relationship between the level of hypertyrosinaemia and the risk of occurrence of keratopathy was seen. For patients on 10 mg nitisinone/day, s‐Tyr levels, at any time during the study, were similar for both unaffected patients (478–1983 μmol/L) and affected patients (609–1264 μmol/L). A comparison of all post‐baseline samples showed a mean (SD) value of 1028.9 (156.8) μmol/L in those who developed keratopathies, and 916.1 (226.9) μmol/L in those with no sign of negative effects on the cornea (Table 3).

**TABLE 3:** s‐Tyrosine and u‐urea_24_ at all post‐baseline visits in patients on 10 mg nitisinone per day with no keratopathy, patients on 10 mg/day who developed keratopathy, and in the latter after reduction to 2 mg/day

Statistic	No keratopathy	Keratopathy patients, 10 mg^a^	Keratopathy patients, after reduction to 2 mg
	N = 59	N = 10	N = 8
*S‐Tyrosine (μmol/L)*			
n	272	23	12
Mean	916.1	1028.9	890.8
SD	226.9	156.8	152.4
Median	885.5	1038.0	901.5
Min	478	609	616
Max	1983	1264	1113
*U‐urea*_*24*_ *(mmol)*			
n	272	23	12
Mean	289.4	333.8	312.2
SD	136.8	102.2	88.7
Median	269.5	334.5	303.3
Min	68	147	186.6
Max	952	575	463.3

*Note*: n = total number of samples collected in the subset at any post‐baseline visit.

^a^ Data for the patient with suspected (unconfirmed) keratopathy are included.

All 10 patients recovered after withdrawal of nitisinone, and eight of them were then prescribed a daily dose of 2 mg/day. (For logistical reasons, this was not possible for the remaining two patients.) The dose reduction resulted in a 13% decrease in s‐Tyr, from 1030.1 (171.2) to 890.8 (152.4) μmol/L. It also resulted in a considerable increase in both u‐HGA_24_ and s‐HGA; u‐HGA_24_ increased 13‐fold and s‐HGA 7‐fold (Tables 3 and 4).

**TABLE 4:** Effect on HGA of reducing the nitisinone dose (N = 8)

Nitisinone dose (mg/day)	u‐HGA_24_ (μmol)		s‐HGA (μmol/L)	
	10	2	10	2
n	23	12	23	12
Mean	153.3	1586.2	0.62	4.38
SD	115.5	953.3	0.75	1.43
Median	136.2	1298.3	0.40	4.10
Min	18.69	690	0.1	2.7
Max	443.52	4064.61	3.5	7.2

*Note*: N: number of patients who changed dose from 10 to 2 mg/day. n: Number of samples collected for the respective dose at any post‐baseline visit.

Further, the patients with keratopathies had higher u‐urea_24_ values compared to non‐affected patients; 338.8 (102.2) mmol vs 289.4 (136.8) mmol. When they were restarted on 2 mg nitisinone/day, they were also reminded of the diet advice and their u‐urea_24_ subsequently decreased by about 6% to 312.2 (88.7) mmol (Table 3).

On the 2‐mg dose, five of the eight patients experienced recurring keratopathies before the end of the study and were then permanently withdrawn (Table S7).

The centre with the lowest average s‐Tyr and u‐urea_24_ values in the nitisinone arm at Month 12 also had the lowest incidence of keratopathies (3.1%), while the centre with the highest s‐Tyr and u‐urea_24_ values showed the highest incidence (12.2% or 14.6% with inclusion of the suspected case) (Table 5).

**TABLE 5:** Mean (SD) s‐tyrosine and u‐urea_24_ at Month 12 by centre and incidence of keratopathies

Centre	S‐ tyrosine (μmol/L)	U‐urea_24_ (mmol)	Incidence of keratopathies
1	1009.2 (237.8)	319.8 (67.7)	6/41 = 14.6%^a^
2	915.4 (169.9)	279.3 (75.5)	3/65 = 4.6%
3	828.6 (187.5)	220.3 (72.4)	1/32 = 3.1%

^a^ Data for the patient with suspected (unconfirmed) keratopathy are included.

## 4 DISCUSSION

Our data have shown that 1 year after receiving the dietary advice (with a reminder at the Month 3 visit), urinary excretion of urea had decreased significantly, confirming compliance with the advice. There was also a statistically significant increase in body weight during the same period. The results varied between centres. At the centre with no overall change in urea there was also no change in body weight, while centres with decreased urea showed increased body weight. Thus, there appears to be a link between decreasing protein intake and increasing body weight, supporting the hypothesis that patients may have compensated the lower intake of protein by eating more carbohydrates and fats. The increase in body weight was seen in the nitisinone‐treated patients, not in the control group. There was also a trend towards a more pronounced decrease in urea excretion in treated patients. This is probably explained by a higher motivation amongst the latter to follow the dietary advice because of the risk of developing keratopathies.

In the untreated control patients, the decrease in urea resulted in a slightly decreased excretion of HGA, with a good correlation between individual data for the two, which is expected since they are both products of protein from the same meals. For unknown reasons, the average s‐HGA actually increased numerically in the control patients, despite a slight decrease in urea, and the correlation between change in s‐HGA and change in u‐urea_24_ was not as good as for u‐HGA_24_ vs u‐urea_24_. Serum samples were collected at different times relative to the urine sampling at the different centres which has probably contributed to the larger variability. Especially if s‐HGA was measured before u‐urea, the two are not products of the same food intake and may therefore show a worse correlation.

It is well‐known that high s‐Tyr may cause precipitation of tyrosine on the cornea, leading to ocular discomfort (pain, red eyes, tearing) and eventually to keratopathies.^9^ In our study, 10 of the 69 treated patients developed keratopathies (confirmed or suspected), and they did so within the first three years of treatment. On the other hand, 52 nitisinone‐treated patients completed the 4‐year study without any tyrosine‐related adverse events, despite similar, and in many cases higher, tyrosine concentrations. It is not known what factors make some patients develop keratopathies, while the majority are unaffected by the hypertyrosinaemia inevitably caused by nitisinone. Further studies are needed to identify the causal mechanisms and concomitant precipitating factors for this, which is also of importance in the management of nitisinone‐treated patients with hereditary tyrosinaemia type 1 (HT‐1).

Data in patients with HT‐1 are mainly in the paediatric population. In those patients it is recommended to keep s‐Tyr below 500 μmol/L, or even as low as 200‐400 μmol/L up to the age of 12 years.^12^ Except for the study by Introne et al,^4^ in which 20 patients with AKU received nitisinone for three years, there is no previous experience with long‐term nitisinone in adult patients not born with tyrosinaemia. The Introne study used no protein restrictions, since adult compliance with such a diet was considered unlikely, published reports in HT‐1 show no correlation between plasma tyrosine concentrations and occurrence of corneal changes,^13^, ^14^, ^15^ and every case of nitisinone‐related keratopathy was reversible upon stopping nitisinone treatment.^13^, ^14^ Only one of the patients in the Introne study, with a lower‐than‐average s‐Tyr level, developed keratopathy. We therefore used a similar approach, but with an introduction of general advice to lower protein intake. Like earlier authors we found no direct link between s‐Tyr levels and keratopathies and many unaffected patients had extremely high s‐Tyr values throughout the study. Despite this, the risk of developing keratopathies seems to increase somewhat with increasing s‐Tyr. On average, the affected patients also had a higher intake of protein than the unaffected ones, as seen from the urinary excretion of urea. The highest incidence of this unwanted effect was seen at the centre which, on average, had the highest s‐Tyr levels and no decrease in u‐urea_24_. The advice to decrease protein intake has thus possibly lowered the risk of developing keratopathies. But this seems to have been followed by an increased risk of body‐weight gain.

In the eight patients who decreased the daily dose from 10 to 2 mg (a decrease of 80%), after recovering from keratopathies, s‐Tyr decreased by about 13%, while s‐HGA and u‐HGA_24_ increased approximately 7‐ and 13‐fold, respectively. This difference in response, between tyrosine and HGA, to different doses of nitisinone agrees with our earlier findings^16^ and shows that decreasing the dose is not the optimum way of reducing s‐Tyr as it has a larger negative effect on the treatment of the disease by resulting in higher HGA levels. It should also be noted that a 6% decrease in protein intake (u‐urea_24_) after restarting treatment on 2 mg/day also contributed to the decrease in s‐Tyr, and thus the actual effect of the dose reduction on s‐Tyr was even smaller, possibly only about 7%.

Five of the eight patients who restarted treatment on 2 mg nitisinone/day experienced recurring keratopathy. This supports the view that dose reduction is not the solution for patients prone to develop keratopathies.

It should be noted that while the observed body‐weight changes and keratopathies are the result of daily protein intake and s‐Tyr levels over a longer time, the measurements in serum and urine of urea, HGA and tyrosine were made on only one single day during the yearly visits to the clinic. Thus, rather large intraindividual variability in these must be expected, depending on day‐to‐day changes in amount and kind of food ingested. Furthermore, at the clinic visits the food eaten may not be representative of the food normally eaten at home. It has therefore not been possible to show good correlations between, for example, individual changes in u‐urea and changes in body weight. A pattern is instead best seen when data are pooled by centre.

A recent study shows that patients with AKU (not treated with nitisinone) have lower muscle mass and higher body‐fat percentage than predicted. They were classified as clinically under‐nourished due to a chronically protein‐restricted diet. AKU patients are often recommended a low‐protein diet to reduce HGA production. But there is no evidence that this has any effect on disease progression.^17^ The protein restriction introduced in our study led to a statistically significant decrease in u‐HGA_24_ in the untreated patients by about 8% on average at Month 12, which cannot be assumed to be of any clinical relevance, especially as there was a simultaneous (unexplained) 6% increase in s‐HGA. For comparison, treatment with nitisinone 10 mg/day reduced u‐HGA_24_ and s‐HGA by, on average, 99.7% and 98.8%, respectively.^8^

The SONIA 2 study was not designed to investigate the effect of a protein‐restricted diet on either keratopathies or body weight. The results presented here are post‐hoc observations that we wanted to explore further to investigate the unexpected finding of weight increases in the nitisinone‐treated group. Our only approximate measure of protein intake was the urinary excretion of urea, which was not originally intended for this use. A more accurate estimation of protein (or nitrogen) intake requires more complex measurements and calculations.^18^, ^19^

Our data support the hypothesis that decreased protein intake may have caused the weight gain. This is in line with the well‐known fact that high‐protein diets reduce body weight by increasing satiety and energy expenditure and decreasing energy intake.^20^, ^21^ It is likely that the opposite mechanisms were responsible for the weight gain seen especially in the nitisinone group. Weight gain in AKU patients is undesirable due to the potential effect on enhancing the connective tissue damage, such as in tendons, ligaments, joints and spine, in addition to being an independent risk factor for the development and progression of type 2 diabetes, hypertension, cardiovascular disease, chronic kidney disease, and cancer.^22^, ^23^ Limitation of weight gain will therefore be an important goal if protein is restricted in connection with the use of nitisinone.

### 4.1 Conclusions

An increase in body weight was seen in the nitisinone‐treated group, but not observed in untreated controls, probably due to the treated patients being more compliant with the low‐protein diet, which was applied without an amino acid supplement. These increases in body weight are undesirable in an already generally overweight patient population. The slight reduction in protein intake may possibly have reduced the risk of developing keratopathies. On the other hand, most nitisinone‐treated patients did not show any signs of corneal reactions, despite high s‐Tyr at levels overlapping those in affected patients.

Dietary restrictions have a great impact on the individual's quality of life, often affecting the rest of the family as well and should therefore not be unnecessarily introduced. Any restrictions should be weighed against both the possible benefits and negative impact on quality of life. If introduced, this should be done in consultation with a dietician. Amino acid supplements, deficient in tyrosine and phenylalanine, may be needed, and the patient's nutritional status should be monitored to avoid malnutrition and obesity.

## CONFLICT OF INTEREST

Birgitta Olsson: Former employee of Sobi, holder of Sobi shares; Lakshminarayan Ranganath: Received consultancy fees from Sobi; Jean‐Baptiste Arnoux: contracted as expert witness on nitisinone treatment in alkaptonuria by Sobi; Mattias Rudebeck: Employee of Sobi, holder of Sobi shares; and Richard Imrich and Anna Milan report no conflicts of interest.

## AUTHOR CONTRIBUTIONS

Birgitta Olsson: Contributed to the study design and data analysis, and, as main author of the manuscript, wrote the original draft. As guarantor for the article, she accepts full responsibility for the work, has access to the data, and controlled the decision to publish. Lakshminarayan Ranganath, Jean‐Baptiste Arnoux, Anna Milan, Richard Imrich: Contributed to the study design, study conduct, data analysis and writing the manuscript, and approved the final manuscript. Mattias Rudebeck: Contributed to the study design, data analysis and writing the manuscript, and approved the final manuscript.

## ETHICS STATEMENT

Independent ethics committees at each centre approved the study. All patients provided written informed consent prior to inclusion in the study.

## Supporting information


**Supplementary Table S1** Demographic data, by treatment group, for patients with data at Month 12
**Supplementary Table S2** Number of patients per BMI category
**Supplementary Table S3** Mean (SD) changes in u‐urea_24_ and body weight from baseline to Month 12, by centre (Patients with data at both visits, N = 127)
**Supplementary Table S4** Mean (SD) changes in u‐HGA_24_ from baseline to Month 12, by treatment and centre
**Supplementary Table S5** Mean (SD) changes in s‐HGA from baseline to Month 12, by treatment and centre
**Supplementary Table S6** Mean (SD) changes in s‐tyrosine from baseline to Month 12, by treatment and centre
**Supplementary Table S7** Time to development of eye disorders leading to temporary or permanent withdrawal of nitisinone

**Supplementary Figure S1** Changes in bodyweight, HGA and tyrosine vs changes in u‐urea_24_, from Baseline to Month 12

## Data Availability

Data access could be granted in response to qualified research requests. Deidentified individual participant data, for patients with separate consent signed for this purpose, could be made available to researchers. Data would be shared on the basis of the scientific merit of the proposal (ie, the proposal should be scientifically sound, ethical, and have the potential to contribute to the advancement of public health) and the feasibility of the research proposal (ie, the requesting research team must be scientifically qualified and have the resources to conduct the proposed project). The data files would exclude data dictionaries that require user licenses. Data requestors would need to sign a data access agreement.
