# Supplementary material for: Effects of a protein‐restricted diet on body weight and serum tyrosine concentrations in patients with alkaptonuria
Source: JIMD Rep. 2021 Nov 9;63(1):41–9. doi: 10.1002/jmd2.12255 (PMC8743336; doi:10.1002/jmd2.12255)
Supplement: Supplementary file 1 — Supplementary Table S1 Demographic data, by treatment group, for patients with data at Month 12 Supplementary Table S2 Number of patients per BMI category Supplementary Table S3 Mean (SD) changes in u‐urea24 and body weight from baseline to Month 12, by centre (Patients with data at both visits, N = 127) Supplementary Table S4 Mean (SD) changes in u‐HGA24 from baseline to Month 12, by treatment and centre Supplementary Table S5 Mean (SD) changes in s‐HGA from baseline to Month 12, by treatment and centre Supplementary Table S6 Mean (SD) changes in s‐tyrosine from baseline to Month 12, by treatment and centre Supplementary Table S7 Time to development of eye disorders leading to temporary or permanent withdrawal of nitisinone [file JMD2-63-41-s002.docx]

**Supplementary Table 1 Demographic data, by treatment group, for patients with data at Month 12**

|  |  | Control (N=63) | Nitisinone (N=66) | Total (N=129) |
| --- | --- | --- | --- | --- |
| Age (years) | Mean | 47.1 | 48.9 | 48.0 |
|  | SD | 9.7 | 10.9 | 10.3 |
|  | Median | 48.0 | 50.0 | 49.0 |
|  | min | 27.0 | 26.0 | 26.0 |
|  | max | 63.0 | 67.0 | 67.0 |
| Body weight (kg) | Mean | 74.3 | 75.1 | 74.7 |
|  | SD | 15.6 | 14.8 | 15.1 |
|  | Median | **74.0** | 75.5 | 74.0 |
|  | min | 46.0 | 36.0 | 36.0 |
|  | max | 122.0 | 110.0 | 122.0 |
| BMI (kg/m^2^) | Mean | 26.3 | 27.0 | 26.7 |
|  | SD | 4.5 | 4.5 | 4.5 |
|  | Median | 25.9 | 26.8 | 26.1 |
|  | min | 18.2 | 17.5 | 17.5 |
|  | max | 41.2 | 39.0 | 41.2 |
| Sex, n (%) | Male | 37 (58.7) | 44 (66.7) | 81 (62.8) |
|  | Female | 26 (41.3) | 22 (33.3) | 48 (37.1) |

SD: Standard deviation

**Supplementary Table 2 Number of patients per BMI category**

|  | **Control (N=63)** | **Nitisinone (N=66)** | **Total (N=129)** |
| --- | --- | --- | --- |
| **BMI category** | **n (%)** | **n (%)** | **n (%)** |
| Underweight BMI < 19 kg/m^2^ | 1 (1.59) | 3 (4.55) | 4 (3.10) |
| Normal BMI 19 - <25 kg/m^2^ | 25 (39.68) | 18 (27.27) | 43 (33.33) |
| Overweight  BMI 25 - <30 kg/m^2^ | 27 (42.86) | 29 (43.94) | 56 (43.41) |
| Obese BMI > 30 kg/m^2^ | 10 (15.87) | 16 (24.24) | 26 (20.16) |

**Supplementary Table 3 Mean (SD) changes in u-urea_24_ and body weight from baseline to Month 12, by centre (Patients with data at both visits, N=127)**

| **Centre (n)** | **Baseline** | **Month 12** | **Change** | **Change (%)*** | **p-value** |
| --- | --- | --- | --- | --- | --- |
| **U-urea_24_ (mmol)** | | | | | |
| 1 (35) | 322.2  (97.6) | 322.1  (74.9) | -0.1  (85.1) | 5.64  (29.29) | 0.995 |
| 2 (62) | 328.6  (18.8) | 288.2 (152.8) | -40.4  (92.5) | -8.69 (27.07) | 0.001 |
| 3 (30) | 283.6  (74.4) | 234.4  (85.0) | -49.2  (81.9) | -15.20 (27.34) | 0.003 |
| Total  (127) | 316.2  (92.0) | 284.8  (89.4) | -31.4  (89.6) | -6.28 (28.63) | 0.0001 |
| **Body weight (kg)** | | | | | |
| 1 (34) | 71.8  (12.7) | 71.7  (13.0) | -0.1  (3.7) | -0.03  (5.05) | 0.926 |
| 2 (64) | 79.6  (14.9) | 81.3  (14.6) | 1.7  (3.7) | 2.14  (4.27) | 0.0004 |
| 3 (31) | 68.1  (15.1) | 69.9  (16.7) | 1.8  (4.2) | 2.50  (5.50) | 0.022 |
| Total  (129) | 74.7  (15.1) | 76.0  (15.5) | 1.3  (3.9) | 1.77  (4.85) | 0.0003 |

* Based on individual % changes

**Supplementary Table 4 Mean (SD) changes in u-HGA_24_ from baseline to Month 12, by treatment and centre**

| **Control** | | | | | **Nitisinone** | | | | |
| --- | --- | --- | --- | --- | --- | --- | --- | --- | --- |
| **Centre (n)** | **Baseline (µmol)** | **Month 12 (µmol)** | **Change (µmol)** | **Change (%)*** | **Centre (n)** | **Baseline (µmol)** | **Month 12 (µmol)** | **Change (µmol)** | **Change (%)*** |
| 1 (16) | 37746 (8996) | 34887 (10488) | -2859 (8720) | -6.30  (24.42) | 1 (18) | 32353 (10322) | 106  (87) | -32247 (10267) | -99.69 (0.17) |
| 2 (32) | 39706 (16123) | 29301 (10349) | -10405 (18185) | -10.78  (67.41) | 2 (31) | 38527 (15348) | 217  (463.2) | -38311 (15289) | -99.42 (1.15) |
| 3 (15) | 26959 (9281) | 23944 (7583) | -3015 (10259) | -3.96  (36.21) | 3 (16) | 29733 (8857) | 74  (69) | -29659 (8830) | -99.76 (0.21) |
| Total  (63) | 36173 (14022) | 29444 (10397) | -6729 (14889) | -8.02 (52.16) | Total  (65) | 34653 (13111) | 151  (328) | -34502 (13044) | -99.58  (0.81) |

* Based on individual % changes

P-values, change in control group: Overall: <0.001; Centre 1: 0.209; Centre 2: 0.003; Centre 3: 0.274

P-values, change in nitisinone group: <0.001; Centre 1: <0.001; Centre 2: <0.001; Centre 3: <0.001

**Supplementary Table 5 Mean (SD) changes in s-HGA from baseline to Month 12, by treatment and centre**

| **Control** | | | | | **Nitisinone** | | | | |
| --- | --- | --- | --- | --- | --- | --- | --- | --- | --- |
| **Centre (n)** | **Baseline (µmol/L)** | **Month 12 (µmol/L)** | **Change (µmol/L)** | **Change (%)*** | **Centre (n)** | **Baseline (µmol/L)** | **Month 12 (µmol/L)** | **Change (µmol/L)** | **Change (%)*** |
| 1 (15) | 29.94  (8.05) | 27.77  (7.63) | -2.09 (7.28) | -3.83 (27.92) | 1 (17) | 35.13 (10.43) | 1.51  (2.80) | -33.63 (11.37) | -94.90  (9.04) |
| 2 (32) | 27.00  (9.08) | 28.88 (11.32) | 1.88  (9.40) | 10.70  (41.19) | 2 (32) | 28.29 (11.82) | 0.34  (0.28) | -27.95 (11.81) | -98.69 (1.22) |
| 3 (15) | 25.78  (7.72) | 25.49  (6.19) | -0.29  (6.96) | 5.27  (37.82) | 3 (16) | 29.61  (9.27) | 0.28  (0.13) | -29.33 (9.25) | -99.03 (0.49) |
| Total  (62) | 27.46  (8.53) | 27.79  (9.44) | 0.39 (8.42) | 5.87 (37.48) | Total  (65) | 30.27 (11.48) | 0.43  (0.72) | -29.84 (11.56) | -98.34 (3.10) |

* Based on individual % changes

P-values, change in control group: Overall: 0.716; Centre 1: 0.284; Centre 2: 0.268; Centre 3: 0.873

P-values, change in nitisinone group: <0.001; Centre 1: <0.001; Centre 2: <0.001; Centre 3: <0.001

**Supplementary Table 6 Mean (SD) changes in s-tyrosine from baseline to Month 12, by treatment and centre**

| **Control** | | | | | **Nitisinone** | | | | |
| --- | --- | --- | --- | --- | --- | --- | --- | --- | --- |
| **Centre (n)** | **Baseline (µmol/L)** | **Month 12 (µmol/L)** | **Change (µmol/L)** | **Change (%)*** | **Centre (n)** | **Baseline (µmol/L)** | **Month 12 (µmol/L)** | **Change (µmol/L)** | **Change (%)*** |
| 1 (16) | 63.0  (11.2) | 61.1  (18.7) | -1.9  (15.8) | -3.03 (24.40) | 1 (18) | 63.6  (13.9) | 1009.2 (237.8) | 945.6 (236.0) | 1561.5 (575.6) |
| 2 (32) | 73.1  (13.0) | 70.7  (20.1) | -2.4  (13.6) | -3.89 (20.12) | 2 (32) | 69.9  (12.0) | 915.4 (169.9) | 845.5 (169.6) | 1246.0 (323.4) |
| 3 (15) | 49.1  (13.6) | 46.5  (15.7) | -2.6  (13.8) | -2.31 (29.49) | 3 (16) | 58.3  (19.3) | 828.6 (187.5) | 770.3 (190.5) | 1479.6 (613.9) |
| Total  (63) | 64.8  (15.9) | 62.5  (21.0) | -2.3  (14.0) | -3.29 (23.31) | Total  (66) | 65.3 (15.1) | 919.9 (202.4) | 854.6 (201.9) | 1388.7 (493.8) |

* Based on individual % changes

P-values, change in control group: Overall: 0.191; Centre 1: 0.631; Centre 2: 0.326; Centre 3: 0.478

P-values, change in nitisinone group: <0.001; Centre 1: <0.001; Centre 2: <0.001; Centre 3: <0.001

**Supplementary Table 7 Time to development of eye disorders leading to temporary or permanent withdrawal of nitisinone**

| Time from start of treatment (days)^a^ | Nitisinone 10 mg/day (N=69) | Nitisinone 2 mg/day ^a^ (N=8) |
| --- | --- | --- |
| n (%) | 10 (14.5) | 5 (62.5) |
| Mean | 485.5 | 445.2 |
| SD | 321.9 | 203.9 |
| Median | 375.5 | 314.0 |
| min | 83 | 277 |
| max | 1075 | 678 |

^a^ For patients who developed symptoms also on the 2 mg/day dose, the time is calculated from start of that dose, following complete recovery after withdrawal of the 10 mg/day dose.

n: Number of patients observed who developed eye disorders leading to temporary or permanent withdrawal of study drug.
