# Supplementary material for: Effects of a protein‐restricted diet on body weight and serum tyrosine concentrations in patients with alkaptonuria
Source: JIMD Rep. 2021 Nov 9;63(1):41–9. doi: 10.1002/jmd2.12255 (PMC8743336; doi:10.1002/jmd2.12255)
Supplement: Supplementary file 2 — Supplementary Figure S1 Changes in bodyweight, HGA and tyrosine vs changes in u‐urea24, from Baseline to Month 12 [file JMD2-63-41-s001.docx]

**FIGURES**

**Supplementary Figure 1 Changes in bodyweight, HGA and tyrosine vs. changes in u-urea_24_, from Baseline to Month 12**

| **a) Change in body weight vs change in  u-urea_24_ (control and treated patients,  n= 127)** | **b) Change in s-tyrosine vs change in  u-urea_24_ (control patients, n = 62)** |
| --- | --- |
| **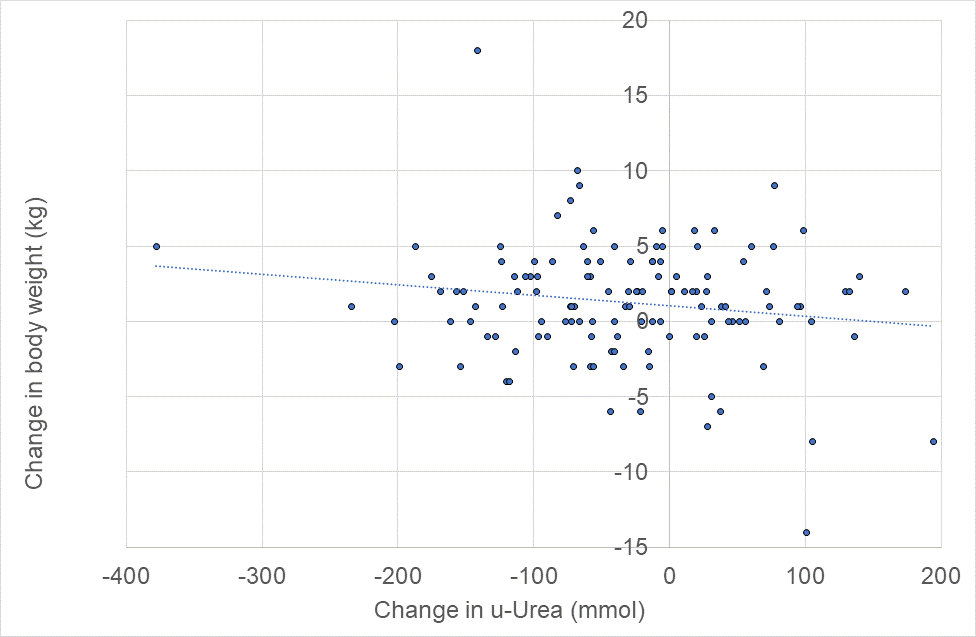** | **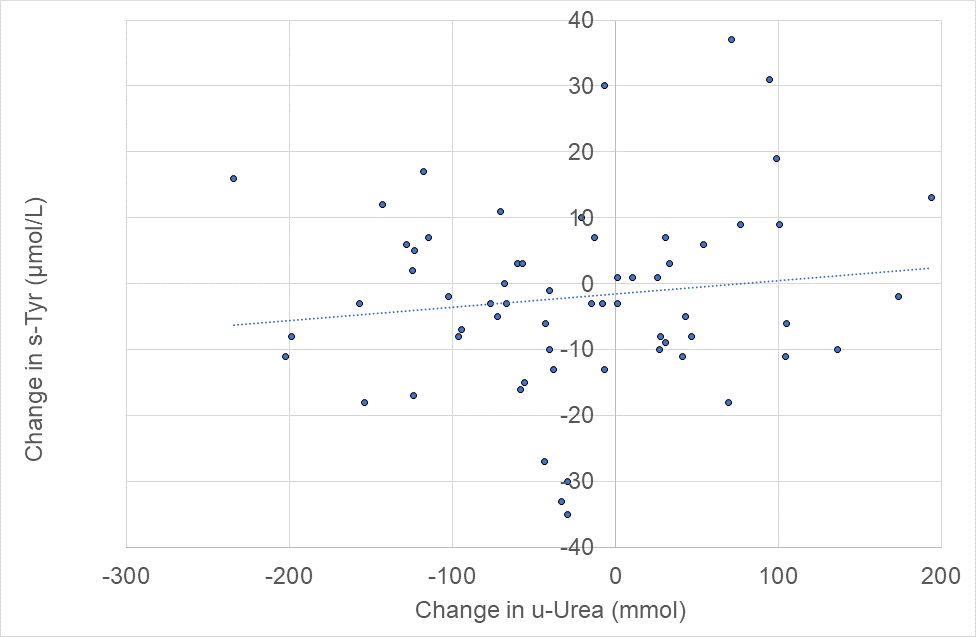** |
| y = -0.0069x + 1.0271 R² = 0.0252 | y = 0.02x - 1.5446 R² = 0.0171 |
| **c) Change in u-HGA_24_ vs change in  u-urea_24_ (control patients, n = 62)** | **d) Change in s-HGA vs change in  u-urea_24_ (control patients, n = 61)** |
| **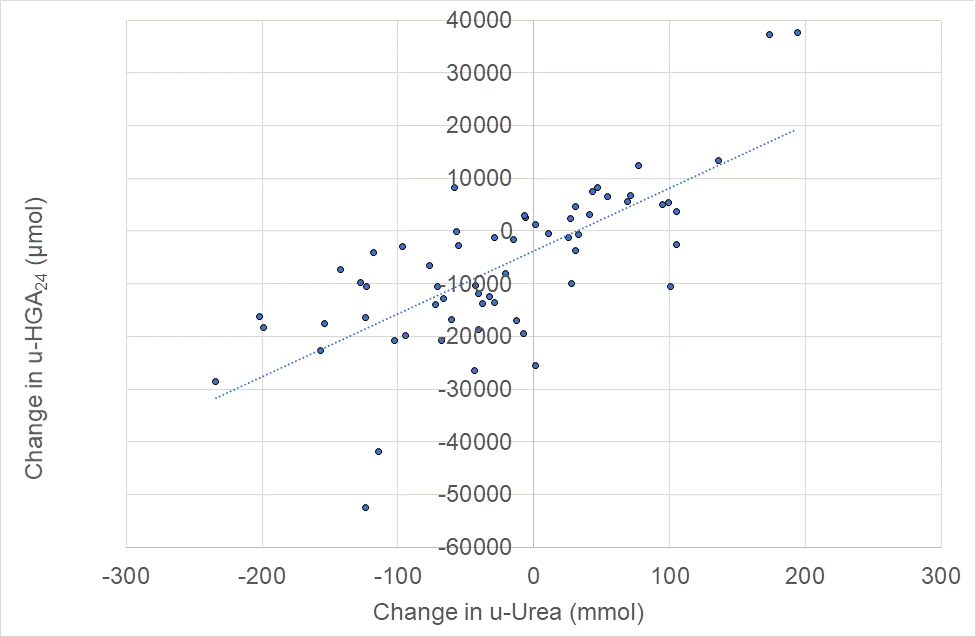** | **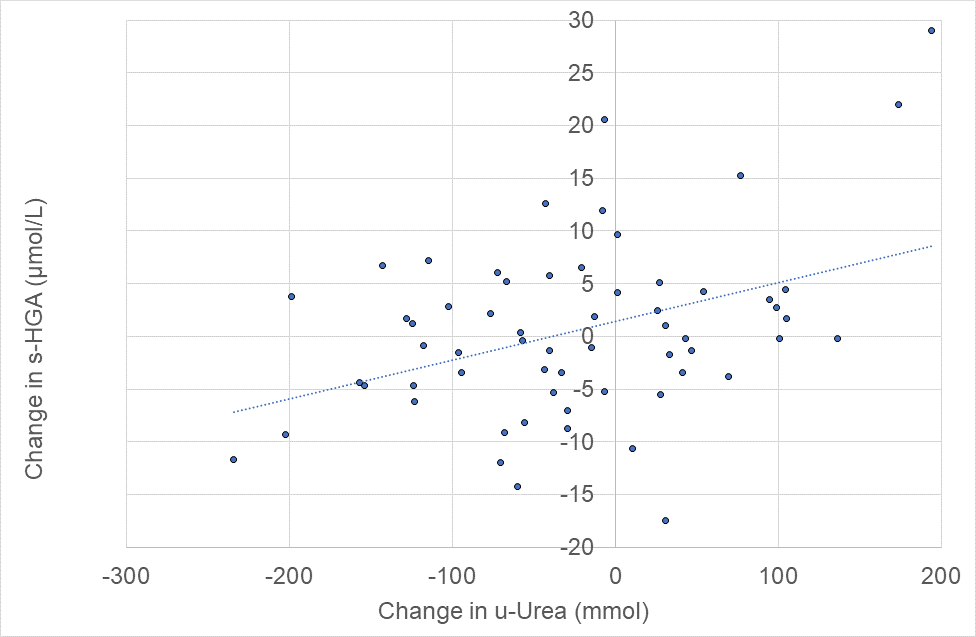** |
| y = 119.41x - 3782.5 R² = 0.5263 | y = 0.0367x + 1.4451 R² = 0.1569 |
